# Management and outcomes of non–small cell lung cancer patients with rapid progression under second‐or‐more‐line immune checkpoint inhibitors: ERORECI study (GFPC 2016‐04)

**DOI:** 10.1002/cam4.2716

**Published:** 2019-11-20

**Authors:** Alain Vergnenegre, Margaux Geier, Florian Guisier, Regine Lamy, Bénédicte Comet, Gwenaelle Le Garff, Pascal Do, Henri Janicot, Hugues Morel, Chantal Decroisette, Michel Andre, Lionel Falchero, Nicolas Paleiron, Isabelle Monnet, the GFPC Team

**Affiliations:** ^1^ CHU Dupuytren UOTC Limoges France; ^2^ Department of Pneumology CHU Morvan Brest France; ^3^ Department of Pneumology, Thoracic Oncology and Respiratory Intensive Care – CHU de Rouen Hôpital Charles Nicolle Rouen France; ^4^ Department of Oncology Centre Hospitalier Bretagne Sud‐Lorient Lorient France; ^5^ Department of Oncology Centre Catalan d'Oncologie Perpignan France; ^6^ Department of Pneumology CH Yves Le Foll Saint‐Brieuc France; ^7^ Centre François Baclesse Caen France; ^8^ Department of Pneumology CHU Hôpital Montpied Clermont Ferrand France; ^9^ Department of Pneumology CHR Orléans Orléans France; ^10^ Department of Pneumology Centre Hospitalier Annecy‐Genevoise Pringy France; ^11^ Department of Pneumology CHU La Réunion Saint‐Denis France; ^12^ Department of Pneumology L'Hôpital Nord‐Ouest Villefranche Sur Saône France; ^13^ Department of Pneumology Hôpital d'Instruction des Armées Sainte‐Anne Toulon France; ^14^ Department of Pneumology Centre Hospitalier Intercommunal Créteil Créteil France

**Keywords:** immune checkpoint inhibitors, non–small cell lung cancer, rapid progression, second‐line treatment

## Abstract

**Background:**

Immune checkpoint inhibitors (ICIs) have been approved as second‐line therapy for advanced non–small cell lung cancers (NSCLCs) progressing after platinum‐based chemotherapy. However, some patients' disease progressed rapidly and sometimes exhibited explosive tumor progression. This descriptive, prospective study aimed to assess the characteristics of nonresponders with rapid progression (RP), defined as progression‐free survival (PFS) ≤2 or 2‐4 months under ICIs.

**Methods:**

This analysis included all consecutive ICI‐treated (second‐or‐more line) patients with RP ≤4 months from 1 September 2016 to 31 August 2017 and compared the clinical characteristics, treatments, and outcomes (overall survival [OS]; responses; PFS, according to treatment line) of NSCLCs that progressed after ≤2 vs 2‐4 months on ICIs.

**Results:**

Comparisons of the 224 (70.2%) patients with ≤2‐month and 95 (29.8%) with 2‐ to 4‐month RP revealed the former had less frequent nonsmokers and ECOG PS = 0, more frequent stage IV disease and higher neutrophil/lymphocyte ratio. Their respective ICI PFS rates were: 1.6 [95% CI: 0.1‐2] and 2.7 [2.0‐4.2] months, with 16.5% and 11.6% having partial responses to first‐ and second‐line therapies post‐ICI chemotherapy. Their respective median OS rates were 6.0 and 9.0 months (*P* ≤ .009). Multivariate analysis retained only PFS of the first‐line therapy pre‐ICI and neutrophil/lymphocyte ratio at ICI onset as being significantly associated with ≤2‐month RP.

**Conclusion:**

In the real‐life setting, NSCLC RP on ICI remains a challenge. New descriptive and analytic studies are needed to identify factors predictive of RP.

## Graphical abstract

Immune checkpoint inhibitors (ICIs) have been approved as second‐line therapy for advanced non–small cell lung cancers (NSCLCs) progressing after platinum‐based chemotherapy. However, some patients' disease progressed before 2 or 4 months—without any response—and sometimes exhibited explosive tumoral progression. This descriptive prospective study aimed to assess the management and clinical outcomes of nonresponders with rapid progression, defined as progression‐free survival ≤2 or 2‐4 months under ICI.

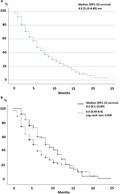

## 1 INTRODUCTION

Immune checkpoint inhibitors (ICIs) represent a major advancement in the management of metastatic non–small cell lung cancers (NSCLCs).^1^, ^2^, ^3^, ^4^, ^5^ As monotherapy, after progression on platinum‐based chemotherapy, they significantly prolong survival with median overall survival (OS) of 9.9 [7.8‐12.4] months.^1^, ^2^, ^5^, ^6^ Nevertheless, rapid progression (RP) characterizes 25% of these tumors, notably during the first 4 months on ICIs or even earlier. Some of those RPs can involve numerous sites, giving the impression of an explosion of metastatic disease.^7^, ^8^ The definitions of RP or hyperprogression are still being debated.^7^, ^8^ That phenomenon has also been observed for other solid tumors.^9^ To define hyperprogression, the tumor growth rate between two computed‐tomography (CT) scans with computer determinations has to be calculated,^7^ which is difficult to do in clinical practice. The RP definition relies on the duration of progression‐free survival (PFS) but no consensus has yet been reached.^8^ According to some studies,^3^, ^4^, ^5^, ^6^ the therapeutic response was influenced by the programmed cell death‐1 ligand (PD‐L1)‐expression rate. However, some NSCLCs undergo early progression, regardless of their PD‐L1 expression rate.^5^

Numerous factors impact the risk of progression: nutritional status^10^, genetic alterations^11^, ^12^, ^13^, ^14^, ^15^ and biological anomalies,^16^ like the neutrophil/lymphocyte ratio,^17^, ^18^, ^19^, ^20^ which has been identified in some studies as also being a predictor of OS or PFS.^21^, ^22^, ^23^, ^24^

The RP definition of varies according to the authors, but the majority of studies retained 2‐ or 4‐months following ICI onset.^25^ Little is known about these patients' characteristics and those of their tumors, therapeutic options post‐ICI and their outcomes.^26^

This study was undertaken to describe the characteristics and management of patients given immunotherapy as second‐or‐more line, whose NSCLCs progressed within the 2 months after starting it, and their outcomes, and compared them to patients whose disease progressed 2‐4 months post‐ICI onset.

## 2 MATERIALS AND METHODS

This multicenter, observational, prospective study included patients >18 years old, managed for stage‐IIIb or‐ IV NSCLC treated with second‐line‐or‐more immunotherapy, whose disease progressed within the 4 months after starting ICI. Almost all patients had progressive disease. Patients not covered by the French National Healthcare System or prisoners could not be included.

The primary outcome criterion was OS after starting ICI, with comparison between patients whose NSCLCs progressed within 2 months (first evaluation) and those whose progression occurred at 2‐4 months (second evaluation). The choice of the first threshold was based on the literature^25^ and the second threshold reflects routine clinical practice, because sometimes ICI is prolonged until the second evaluation, according to the clinical benefit. Secondary criteria were clinical characteristics, PFS on the different treatment lines before ICI and efficacies of post‐ICI therapies.

The information was collected from medical records: clinical and histological findings, biological characteristics (neutrophil and lymphocyte counts) at NSCLC diagnosis and then at ICI onset, treatments before and after immunotherapy. Each PFS was calculated from initiation of the treatment line until the disease progressed, according to RECIST v1.1 criteria, which were assessed by each local investigating team, including a radiologist. No central review was done because this was a real‐world study.

Statistical analyses were computed with StatView v5.0 (SAS Institute Inc). They included descriptive analyses: frequencies of qualitative variables, and means ± standard deviation (SD), medians [95% confidence intervals (CIs)] for quantitative variables. Between‐group comparisons (≤2‐ vs 2‐ to 4‐month RP) used chi^2^ or Fisher's exact tests. Univariate analyses of dichotomized variables used ANOVA. Survival analysis was estimated with Kaplan‐Meier curves. Factors predictive of survival and RP ≤2 vs 2‐4 months were identified with a Cox backward step‐by‐step logistic‐regression model comprised of variables achieving *P* < .25 in univariate ANOVA.

In accordance with French law, the study was approved by Limoges University Ethics Committee on 23 March 2017.

## 3 RESULTS

Between July 2016 and July 2017, 20 GFPC (French Lung Cancer Group) centers prospectively included 319 patients: median age: 64.3 years; 70.8% men; 91.6% smokers or ex‐smokers; 92.9% with Eastern Cooperative Oncology Group performance status (ECOG PS) PS = 0/1, 7.1% PS = 2; predominantly (63.6%) adenocarcinomas and 50.2% with >1 metastatic site at ICI onset (see Table 1 for other characteristics). Because PD‐L1 testing was not generalized in France until September 2017, its status had been obtained for only 9% of the patients. All the patients had received nivolumab, the only ICI available at that time, initially as compassionate therapy, then after its marketing authorization had been obtained. Data about oncogenic drivers were not available for all patients, depending on the study period in France. However, 82 (27.5%) patients had Kirsten rat‐sarcoma viral oncogene mutations, with the other biological markers being scarce: nine epidermal growth factor receptor mutations, four the v‐*RAF* murine sarcoma viral oncogene homolog‐B mutations, four human epidermal growth‐factor–receptor‐2 overexpressions, and four tyrosine kinase c‐*Met* protooncogene mutations.

**Table 1:** Characteristics of the 319^a^ NSCLC patients at diagnosis, with univariate analysis comparison of the two RP groups

Characteristic	Entire cohort (N = 319)		RP < 2 mo (N = 224)		RP 2‐4 mo (N = 95)		*P*
Age: median (range), y	64.3 (25‐89)		65.0 (25‐87)		64.2 (46‐87)		.21
Sex							
Male	226	70.8%	158	70.5%	68	71.6%	.35
Female	93	19.6%	66	29.5%	27	28.4%	
Smoking status							
Nonsmoker	27	8.4%	16	7.2%	11	12.2%	.06
Smoker	138	43.1%	100	46.0%	32	39.6%	
Ex‐smoker	155	48.5%	106	47.8%	47	48.9%	
ECOG PS							
0	130	46.7%	87	44.6%	43	51.2%	.011
1	129	46.2%	94	48.2%	35	41.7%	
≥2	20	6.1%	14	7.2%	6	7.1%	
Stage at diagnosis							
I‐II‐III	80	28.2%	66	29.5%	14	16.3%	.03
IV	229	71.8%	157	70.5%	72	83.7%	
Histology							
Squamous cell	94	29.4%	62	27.7%	32	33.7%	.53
Adenocarcinoma	203	63.6%	144	64.2%	59	62.1%	
Undifferentiated	22	7.0%	18	8.1%	4	4.2%	
No. of metastatic sites							
≤1	114	49.8%	79	50.6%	35	47.9%	.33
>1	115	50.2%	77	49.4%	38	52.1%	
Metastatic site(s) at diagnosis							
Lung	77	20.8%	50	20.2%	27	21.9%	.63
Brain	51	13.8%	31	12.5%	20	16.7%	
Nodes	32	8.6%	25	10.7%	7	5.7%	
Liver	45	12.2%	32	12.9%	13	10.6%	
Bones	86	23.2%	57	23.1%	29	23.6%	
Skin	10	2.7%	5	2.0%	5	4.1%	
Others	69	18.6%	47	19.0%	22	17.9%	
No. of lines before ICI							
1	203	63.7%	147	65.9%	55	59.8%	.94
2	82	25.8%	54	24.2%	28	29.5%	
3	34	10.6%	22	9.9%	12	12.7%	
No. of ICI infusions, median (range)	4 (1‐10)		3.99 (1‐10)		5 (1‐8)		.0001
NLR, mean ± SD							
NLR1 at ICI onset (n = 231)	7.79 ± 21.1 G/L		7.83 ± 21.31 G/L		7.69 ± 20.6 G/L		.01
NLR2 at progression on ICI (n = 226)	10.90 ± 46.1 G/L		8.13 ± 13.43 G/L		12.72 ± 83.42 G/L		.06
NLR2 − NLR1: (n = 193)	−0.14 ± 19.44 G/L		0.42 ± 18.83 G/L		−1.58 ± 20.91 G/L		.92

Note

Results are expressed as n (%) unless stated otherwise.

Abbreviations: ECOG PS, Eastern Cooperative Oncology Group performance status; G/L: giga/liter; ICI, immune checkpoint inhibitor; NLR1/2, neutrophil/lymphocyte ratio at ICI initiation/ICI progression; NLR1 − NLR2, difference between ratios; NSCLC, non–small cell lung cancer; RP, rapid progression; SD, standard deviation.

a Patient numbers vary as a function of the number of missing data.

Before starting ICIs, all but three patients had received first‐line platinum‐based chemotherapy: doublet for 269/319 (84.3%), triplet (including bevacizumab) for 47/319 (14.7%) and 171 received maintenance chemotherapy. Among the 82 (25.7%) patients given pre‐ICI second‐line therapy, 38 received bitherapy, eight tritherapy and 36 monotherapy. Finally, among the 34 who received pre‐ICI third‐line therapy, 22 were given monotherapy, three tritherapy and nine targeted therapy. Notably, 63.7% of ICI treatments were second‐line therapy. The mean pre‐ICI neutrophil/lymphocyte ratio was 7.8 ± 21. At the beginning of ICI treatment, 18 (5.6%) patients received corticosteroids: eight (2.5%) at a dose ≤ 10 mg and 10 at a dose ≥15 mg to control brain metastases.

Among the 319 patients included in the cohort, 224 experienced RP within ≤2 months and 95 between 2 and 4 months. Adverse events occurred in 52 (16%) patients: grade 1‐2 for 35 (10.9%), without any consequences on ICI treatment; grade 3 for 12 (5%; three digestive, three hepatic, two respiratory, four cutaneous toxicities), treated with dose reduction or temporary treatment stoppage; one (0.3%) grade‐4 digestive toxicity with treatment interruption.

The cohort's PFS was 1.8 [95% CI: 0.2‐4.2] months. This short duration is explained by cohort constitution (all patients had progressive disease). Among the 319, 167 progressed within 2 months after starting ICI; their PFS lasted 1.6 [95% CI: 0.2‐2] months vs 2.7 [95% CI: 2.2‐4.2] months for those with RP 2‐4 months. The more rapid progressors had significantly worse ECOG PS, more advanced stage NSCLCs, and higher neutrophil/lymphocyte ratios at ICI onset and lower rates at the end of ICIs. Their PFS was significantly shorter on first‐ and second‐line therapies before ICI than for those with RP 2‐4 months. The cohort received a median of 4 [95% CI: 1‐10] immunotherapy cycles: 3.99 and 5 for RP ≤ 2 and 2‐4 months, respectively.

**Figure 1:**
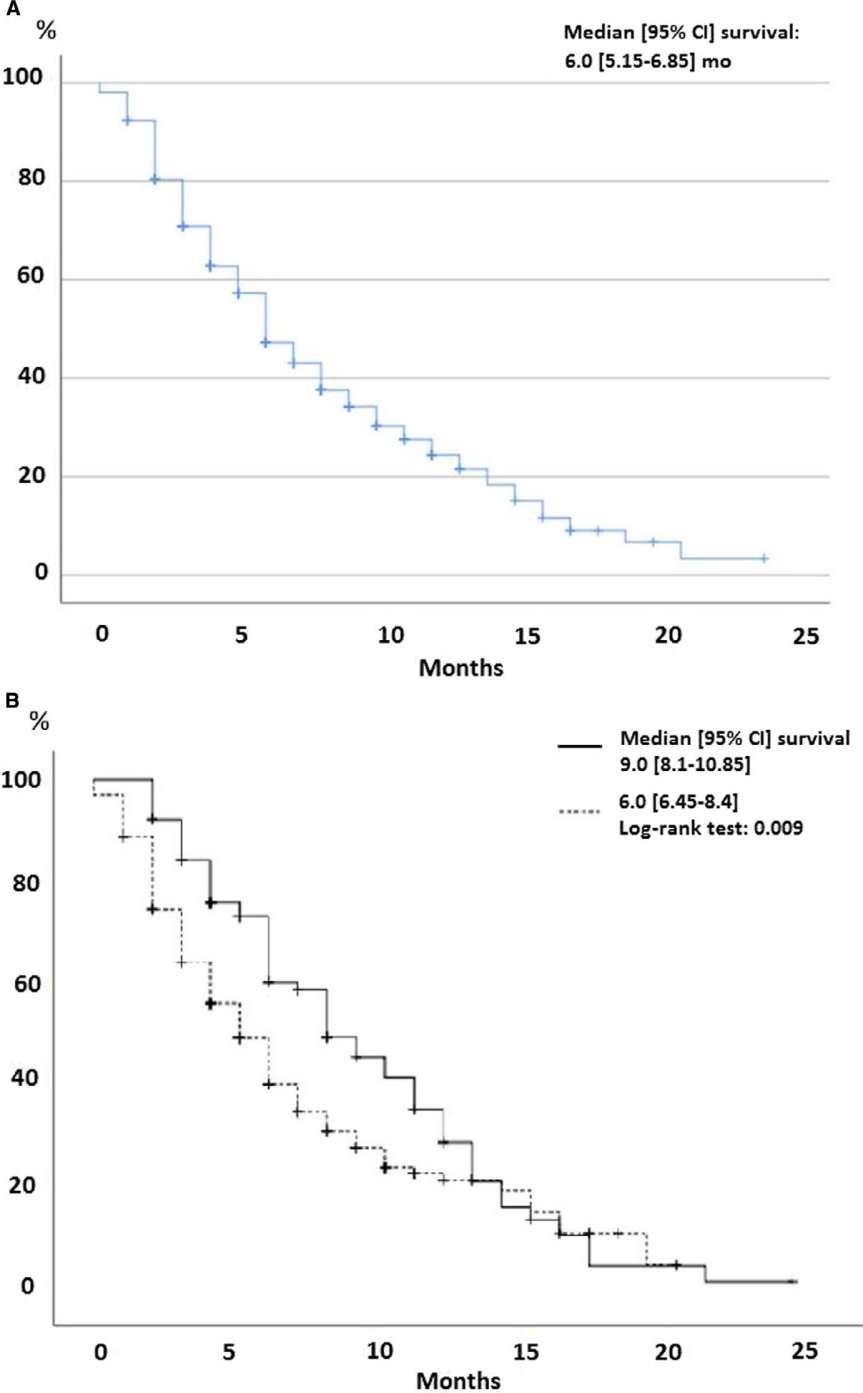
Kaplan‐Meier survival curves (A) post immune checkpoint inhibitors (ICI) for the entire study cohort or (B) according to rapid progression after ≤2 (dotted line) or 2 to 4 months (solid line) on ICIs

Progression‐free survival rates on first‐, second‐, and third‐line therapies, as a function of RP on ICI, are reported in Table 2. Those results clearly showed that PFS for the two first lines pre‐ICI was longer for 2‐ to 4‐month RP group than those progressing within ≤2 months. PFS3 is difficult to analyze because of the small number of patients. Post‐ICI, 212 (66.5%) and 69 (21.6%) patients, respectively, were given first‐ and second‐line treatments (Table 3). Their respective response and control rates were 16.7% and 38.2%, and 11.6% and 27.5%. Median OS from ICI onset was 6 [95% CI 5.15‐6.85] months (Figure 1A), and differed significantly between RP ≤ 2 (6 [95% CI 6.45‐8.4]) and 2‐4 (9 [95% CI 8.17‐10.85] months; *P* < .009) (Figure 1B).

**Table 2:** PFS on successive treatment lines before immune checkpoint inhibitors (ICIs) according to RP on ICI

Pre‐ICI line	N	RP on ICI				*P*
		≤2 mo		2‐4 mo		
		Median	Range	Median	Range	
PFS1	314	5.8	0.3‐25.0	9.0	0.7‐34.0	.001
PFS2	93	2.8	0.56‐21.2	8.3	0.7‐25.8	.018
PFS3	29	3.0	1.6‐21	3.63	1.6‐18.6	.93

Abbreviations: FPFS, progression‐free disease; PFS1/2/3: PFS on first‐, second‐ or third‐line treatment before ICI(s); RP, rapid progression.

**Table 3:** Post‐ICI treatments for the 319 metastatic NSCLC patients

Parameter	Number	%		
Number of post‐ICI treatment lines				
0	107	33.5		
1	212	66.5		
2	69	21.6		
1st‐line post‐ICI chemotherapy	204			
Pemetrexed	18			
Gemcitabine	31			
Docetaxel	76			
Paclitaxel	44			
Vinorelbine	3			
Targeted therapy	23			
Others	17			
Response to 1st‐line post‐ICI treatment	204			
Partial response	34	16.7	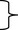	38.2% controlled disease
Stable disease	44	21.5		
Progressive disease	68	33.3	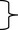	61.8% progressors
Early deaths, deemed progressors	58	28.4		
2nd‐line post‐ICI chemotherapy	69			
Pemetrexed	5			
Gemcitabine	17			
Docetaxel	3			
Paclitaxel	11			
Vinorelbine	3			
Targeted therapy	17			
Others	13			
Response to post‐ICI 2nd‐line chemotherapy	69			
Partial response	8	11.6	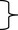	27.5% controlled disease
Stable disease	11	15.9		
Progressive disease	26	37.7	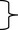	72.5% progressors
Early deaths	24	34.8		

Abbreviations: ICI, immune checkpoint inhibitor; NSCLC, non–small cell lung cancer.

Multivariate analysis retained only pre‐ICI PFS after first‐line therapy and the neutrophil/lymphocyte ratio at ICI onset as being significantly associated with RP ≤ 2 months (Table 4).

**Table 4:** Multivariate backward step‐by‐step Cox regression model of rapid progression ≤2 mo on immune checkpoint inhibitor

Variable	Hazard ratio	95% Confidence interval	*P*
NLR1	1.023	1.001‐1.045	.037
PFS1	0.997	0.995‐0.999	.005

Note

Variables included in the model: age, smoking status, Eastern Cooperative Oncology Group performance status, number of metastatic sites, progression‐free survival on the 1st‐line treatment (PFS1), PFS2, NLR1: ratio neutrophil/lymphocyte ratio at ICI onset (NLR1).

## 4 DISCUSSION

This multicenter, observational, prospective study was designed to determine the clinical, biological, and evolutionary characteristics of patients with NSCLC progression and compared as a function of their RP ≤ 2 or 2‐4 months. Its results showed that a shorter PFS after first‐line therapy and marked inflammation at ICI onset were significantly associated with the earlier RP. Those factors were accompanied by shorter survival. However, it was possible to treat these patients after progression on ICI, with some of them achieving satisfactory survival.

Hyperprogression among immunotherapy‐treated patients is a real challenge for oncologists. And its precise mechanisms are still being debated: primary resistance, PD‐1 expression on T‐regulatory cells, compensatory T‐cell exhaustion, modulation of tumor‐promoting cells, aberrant inflammation, and activation of an oncologic pathway.^7^ This investigation concerned patients with metastatic NSCLCs that progressed rapidly, defined as a very short interval between ICI onset and diagnosis of progressive disease.^25^ We did not focus on patients with recently defined hyperprogression,^8^ which requires at least two CT‐scans before starting ICI to evaluate the tumor's doubling rate.^7^, ^9^

The RP definition varies according to authors. Some base it on the tumor‐doubling time,^7^, ^8^, ^9^ whereas others rely on death occurring within the 3 months following ICI onset^27^ or ≥3 nivolumab infusions.^26^ Constantini et al^28^ considered patients to have refractory NSCLCs when their disease progressed after one or two nivolumab injections. Shiroyama et al retained a threshold of 2 months.^25^ Depending on the definition applied, NSCLC RP frequencies ranged from 9% to 20%.^26^ The characteristics of those patients also varied^28^, ^29^ but the majority of them had multisite metastatic disease.

The authors of most studies compared progressor's clinical and biological characteristics to those of responders. Our findings are consistent with those of other studies.^16^, ^18^, ^25^, ^26^, ^29^ Shiroyama et al^25^ found ECOG PS and inflammation to be factors associated with RP, whereas, according to Costantini et al's very recent publications,^26^, ^28^ RP was significantly associated with ECOG PS, bone metastases or short duration of pre‐ICI treatment. Their observations are consistent with ours, showing a significant link between short first‐line therapy PFS before ICI and inflammation at ICI onset as factors discriminating between RP < 2 and 2‐4. In contrast, age, ECOG PS, smoking status, pre‐ICI second‐line therapy PFS, neutrophil/lymphocyte ratio at the end of ICI did not differentiate between the two RP groups.

Our study has several limitations. First, the results were influenced by the chosen RP definition, which could be different with another threshold but there is no consensus for the RP definition. Second, its observational nonrandomized design means data could be missing, notably for certain biological markers. Third, the absence of PD‐L1 status, which prevents exploration of the impact of this parameter on prognosis. Fourth, the multicenter participation meant RP identification was left to the physicians at each site without central review, as in real‐life, routine practice. Last, some data were lacking, like serum albumin or lactate dehydrogenase, which could not be included in the model.

## 5 CONCLUSION

Non–small cell lung cancers RP on ICI remains a very real challenge because of the clinical deterioration it represents. Factors predictive of these NSCLCs need to be specified in the framework of large cohorts, with clinical and biological data, as in ongoing trials (NCT03412058). In addition to clinical trials, new descriptive and analytical studies will be essential once a clear definition of RP is established. Artificial intelligence could allow noninvasive radiomic biomarkers, as recently described,^30^, ^31^ to combine clinical and radiological data.

## CONFLICTS OF INTEREST

M Geier, R Lamy, B Comet, G Le Garff, P Do, H Janicot, H Morel, M Andre, L Falchero, N Paleiron, I Monnet, M Dusselier have no commercial interests to declare. C Decroisette has received honoraria for consultancies and fees for medical conferences from Roche, BMS, Pierre Fabre, Novartis, AstraZeneca, and Boehringer Ingelheim. F Guisier has received honoraria from AstraZeneca, Boehringer Ingelheim, MSD, BMS, and Roche. A Vergnenègre has received honoraria for consultancies and fees for medical conferences from MSD, Hoffman Laroche, BMS, Pierre‐Fabre Oncology, AstraZeneca, and Boehringer Ingelheim.

## Data Availability

I confirm that I have included a citation for available data in my references section, unless my article type is exempt.

## APPENDIX A

### AUTHORS CONTACT DETAILS

Margaux Geier: margaux.geier@gmail.com

Florian Guisier: florian.guisier@gmail.com

Régine Lamy: r.lamy@ch-bretagne-sud.fr

Bénédict Comet: benedictecomet@gmail.com

Gwenaelle Le Garff St Brieuc: gwenaelle.legarff@ch-stbrieuc.fr

Pascal Do: PDO@baclesse.unicancer.fr

Henri Janicot: hjanicot@chu-clermontferrand.fr

Hugues Morel: hugues.morel@chr-orleans.fr

Chantal Decroisette: cdecroisette@ch-annecy.fr

Michel Andre: michel.andre@chu-reunion.fr

Lionel Falchero:LFalchero@lhopitalnordouest.fr

Nicolas Paleiron: nicolas.paleiron@free.fr

Isabelle Monnet: Isabelle.Monnet@chicreteil.fr
